# Novel 3-Amino-2-methylquinazolinone NF-κB Inhibitors: Synthesis and Potential Anti-Inflammatory Function

**DOI:** 10.3390/ijms27146431

**Published:** 2026-07-20

**Authors:** Chrysoula Mikra, Vasiliki Petriki, Evangelos Tsioupros, Eleni Kougioumtzian, Konstantinos Michail, Stella Manta, Barry J. Campbell, Konstantina C. Fylaktakidou, Stamatia Papoutsopoulou

**Affiliations:** 1 Laboratory of Organic Chemistry, Faculty of Chemistry, Aristotle University of Thessaloniki, 54124 Thessaloniki, Greece; chrmikgeo@chem.auth.gr (C.M.); stmanta@chem.auth.gr (S.M.); 2 Department of Biochemistry and Biotechnology, University of Thessaly, 41500 Larissa, Greeceetsioupros@uth.gr (E.T.); elinakoug@yahoo.gr (E.K.); kostantinemix@gmail.com (K.M.); 3 Department of Infection Biology and Microbiomes, Institute of Infection, Veterinary and Ecological Sciences, University of Liverpool, Liverpool L69 3GE, UK; bjcampbl@liverpool.ac.uk

**Keywords:** NF-κB, inflammation, 3-amino-2-methylquinazolinone, transcription

## Abstract

The canonical NF-κB pathway is a major regulator of inflammatory responses, and its persistent activation has been linked to chronic inflammatory diseases and cancer development. It is therefore important to develop effective NF-κB inhibitors that can potentially be utilized in further preclinical studies. In this study, we have synthesized various quinazolinone compounds using novel methodologies. We specifically utilized a two-step protocol comparing an established protocol to a modified alternative procedure and then successfully established a novel one-pot multi-component reaction. All quinazolinones were tested based on their capacity to inhibit tumor necrosis factor-induced NF-κB activation using a commercial transgenic HeLa reporter cell-line. Among the eighteen molecules tested, two compounds affected the NF-κB-dependent luciferase activity, the **5o** (3-amino-6-hydroxy-2-methylquinazolin-4(3*H*)-one) and **10** (*N*-(6-bromo-2-methyl-4-oxoquinazolin-3(4*H*)-yl)-4-nitrobenzamide). Only the **5o** though, bearing OH at R^1^ (6 position), attenuated the production of pro-inflammatory interleukin 6 in lipopolysaccharide-stimulated human primary macrophage cultures. Molecular docking analysis provides supportive computational evidence that **5o** could interact with the p65/DNA complex and therefore could potentially be developed for further studies that aim to inhibit the canonical NF-κB pathway.

## 1. Introduction

**Figure 1:**
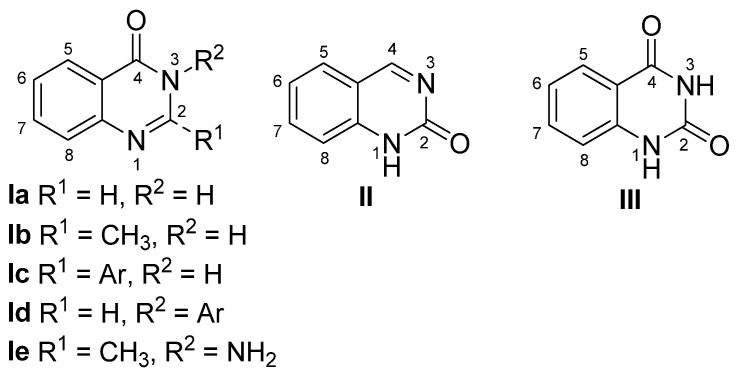
Types of quinazolinones: 4(3*H*)quinazolinone (**Ia**), 2-methyl-4(3*H*) quinazolinones (**Ib**), 2-aryl-4(3*H*)quinazolinones (**Ic**), 3-aryl-4(3*H*)quinazolinones (**Id**), 3-amino-2-methyl-4(3*H*)quinazolinones (**Ie**), 2(1*H*)quinazolinone (**II**) and 2,4(1*H*,3*H*)quinazoline-dione (**III**). Structures are given in their simplest non-substituted forms.

The 4(3*H*)quinazolinone frame is a nitrogen-rich heterocyclic scaffold consisting of an oxidized quinazoline ring and a ketone group [1]. It is ubiquitous in nature, particularly forming the core structure of numerous naturally occurring alkaloids found in plants, animals, and microbes [2,3]. Quinazolinones are classified according to the position of their oxo group to three types: 4(3*H*)quinazolinone (**Ia**), 2(1*H*)quinazolinone (**II**) and 2,4(1*H*,3*H*)quinazoline-dione (**III**); see Figure 1. From a chemical point of view, all quinazolinones can be derivatized at the aromatic ring (positions 5–8), whereas for **I** and **II**, these structures might be additionally substituted at positions 2 or 4, respectively. NH groups might be found substituted in all quinazolinones, whereas tautomerization with the carbonyl gives possibilities for further functionalization. More specifically for frame **I**—which is the targeted scaffold in this study—compounds **Ib**, 2-methyl-4(3*H*) quinazolinones, possess capacity for additional reactivity towards electrophiles and oxidizers on the methyl group, due to neighboring assisted activation. Therefore, the biological importance of both natural and synthetic derivatives of 4(3*H*)quinazolinones (**I**) (referred to as ‘QNZ’ from here on) renders them among the “privileged” medicinal motifs [4,5].

A key advantage in QNZ (**I**) synthesis is that it requires only inexpensive, commercially available reagents such as *o*–amino–benzonitriles, substituted anthranilic acids or esters, benzamides, and *o*–halo–benzoic acids. In addition, there are relatively easy ways to synthesize such frames, including conventional synthesis [6], organocatalytic or solid acid catalyzed approaches [7,8], as well as green chemistry microwave-assisted [9,10,11,12] and light-mediated [13,14] syntheses. In terms of synthetic utility, aryl substituents at 2- and 3- positions of QNZs are key structural domains to facilitate C-H functionalization and annulation (Figure 1, **Ic** and **Id**, respectively) [15]. Finally, 3–amino–2–methyl–QNZs, the compounds of interest herein (Figure 1, **Ie**) are considered a synthon, an “intermediate compound” that allows a variety of groups, such as aromatic and heteroaromatic moieties, to be linked or attached on the quinazolinone heterocyclic ring, usually in the form of a Schiff base or an amide.

3-amino-substituted QNZs and derivatives thereof (Figure 1, **Ie**, R^1^ = any, R^2^ = NH_2_ or NR’R’’) have, among others, antitumor [16,17] and anti-inflammatory activities [18,19] and these functions are shown to be facilitated by inhibition of pathways important for cancer development, such as the DNA-repair enzyme WRN (Werner syndrome) helicase [20], the VEGFR2 (Vascular Endothelial Growth Factor Receptor 2) pathway that promotes angiogenesis [21], or the canonical (classical) NF-κB pathway that drives the inflammatory responses of immune cells [22]. The NF-κB family of transcription factor protein complexes consists of five member subunits, p105/p50, RelA(p65), c-Rel, p100/p52, and Rel-B that form homo- and heterodimers [23]. The predominant NF-κB transcription factor dimer combination involved in canonical pro-inflammatory signaling is the p50:RelA(p65) heterodimer [24]. Dysregulated, constitutive activation of the NF-κB pathway has been observed in chronic inflammatory diseases, such as inflammatory bowel disease and rheumatoid arthritis [25,26]. Moreover, activation of this pathway correlates with various aspects of cancer development, including tumor cell proliferation and survival, metastasis, tumor angiogenesis, and resistance to therapy [27,28]. Each can be attributed to transcriptional regulation of specific NF-κB target genes, such as *PTGS2* encoding cyclooxygenase-2 (COX-2) [29], *IL1B* and *IL6* encoding inflammatory cytokines interleukin 1 (IL-1) [30], and interleukin 6 (IL-6), respectively [31], and key anti-apoptotic genes encoding B-cell lymphoma-2 (Bcl-2) and inhibitor of apoptosis (IAP) family proteins, whose overexpression can prevent tumor cells from undergoing programmed cell death and cell cycle progression [32]. Other key regulated NF-κB specific transcripts include *CCND1* (cyclin D1) and *c-MYC* (regulatory proto-oncogene c-Myc), that can drive uncontrolled tumor cell proliferation and resistance to apoptosis [33]. Therefore, NF-κB is considered an attractive target when designing and developing pharmaceutical drugs based on structures such as quinazolinones and their derivatives [22,34]. We recently identified a novel quinoline molecule (a heterocyclic aromatic nitrogen compound characterized by a double-ring structure consisting of a benzene ring fused to a pyridine ring at adjacent carbon atoms [35]) as having inhibitory activity for the canonical NF-κB pathway [36]. Using a commercial transgenic HeLa cell-line as an NF-κB activation reporter, this novel quinoline, Q3, inhibited NF-κB-induced luciferase, without impacting on cell survival [36].

In this study, we have synthesized 3-amino-2-methyl QNZ compounds via a two-step protocol and then successfully established a novel one-pot multi-component reaction. Several amide derivatives were also synthesized, supporting comparison studies for the role of the free amine on the QNZ scaffold. We then evaluated the in vitro activity of the new compounds as modifiers of the canonical NF-κB pathway, utilizing a human epithelial reporter cell-line, HeLa/NF-κB Luc. We showed that two specific quinazolinones (**5o** and **10**) could inhibit NF-κB-regulated luciferase activity, but only one of these (**5o**) had a significant biological impact, inhibiting the production of the pro-inflammatory IL-6 from human primary macrophage cultures. Moreover, in silico analysis supported that this quinazolinone could potentially interfere with the DNA-binding activity of the p65/NF-κB transcription factor.

## 2. Results

### 2.1. Synthesis of 3-Amino-2-methyl QNZs

**Figure 2:**
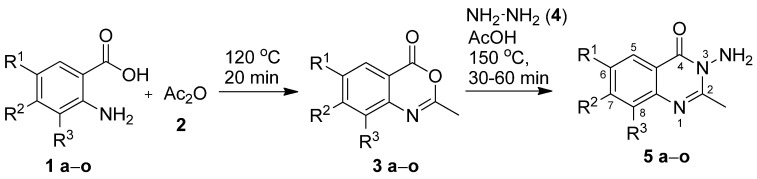
Two-step procedure for the synthesis of 3NH_2_-2methylquinazolinones **5a**–**o** in an autoclave. Numbers outside the structure indicate the numbering of the quinazolinone nucleus.

The synthesis of the compounds used in this study was based on the modification of a previously described protocol performed under microwave irradiation (MW) [12]. As the combined yields in that particular protocol were low, instead of using MW, we designed and performed an alternative two-step procedure using an autoclave with conventional heating, first in acetic anhydride (Ac_2_O) and then in acetic acid (AcOH). Specifically, anthranilic acids **1a**–**o** were each mixed with Ac_2_O (**2**) to form the related benzoxazinones **3a**–**o** upon heating in an autoclave at 120 °C for 20 min (Figure 2). Solid material that formed upon cooling of the reaction mixture was filtrated, washed with petroleum ether, subsequently mixed with hydrazine monohydrate (NH_2_NH_2_, **4**) in AcOH, and heated in an autoclave at 150 °C for 30–60 min to give **5a**–**o** in moderate-to-high yields over two steps, a significant improvement compared to the MW method; see Table 1.

**Figure 3:**
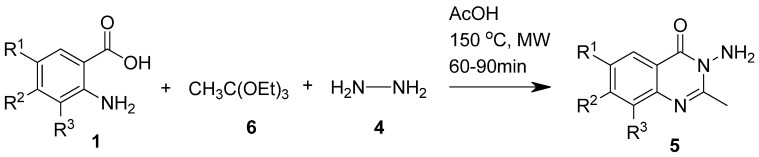
One-pot procedure for the synthesis of 3-NH_2_-2-methylquinazolinones **5a**–**o** under microwave (MW) irradiation-assisted procedure.

We recently reported a one-pot four-component reaction for the synthesis, with very good yields of 2-methyl-3-hydroxyquinazolinone derivatives, upon mixing of anthranilic acid, triethylorthoacetate, hydroxylamine hydrochloride, and pyridine [11]. It was reasonable to adopt the same methodology with hydrazine as an alternative nucleophile. Therefore, we attempted and successfully established a novel one-pot procedure by simply mixing all components **1**, **4**, and **6** (CH_3_C(OEt)_3_; triethyl orthoacetate) to receive compound **5** in good-to-high yields, as shown in Figure 3. Nitro derivatives **5k**,**l** were reluctant to react, as in the case of 3-hydroxy related reactions [11]. Nevertheless, the yields of the products for the rest of the performed reactions increased by 4 to 25%.

The composition of compounds **1**, **3**, and **5** regarding R^1^, R^2^, and R^3^ as well as comparison of the % yields of product **5** between the two-step (autoclave and microwave irradiation) and one-pot microwave-assisted procedures is shown in Table 1.

**Figure 4:**
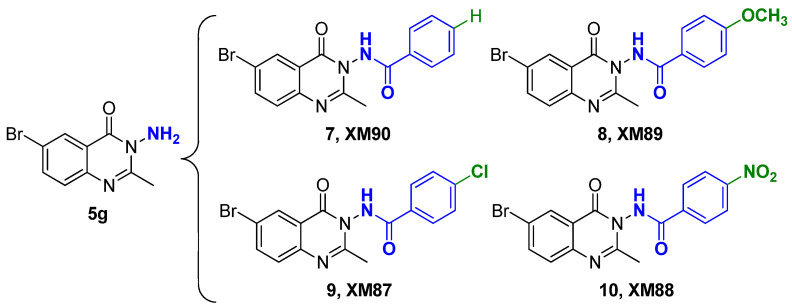
Structures of benzoylated derivatives **7**–**10** of 3-amino-5-bromo-2-methylquinazolinone **5g**. The blue font color indicates derivatization on the free amino group of **5g**. The green color is used to identify the different substituents on the aromatic group.

Evaluation of the importance of the free NH_2_ group in the activity of the compounds **5a**–**o** towards NF-κB was clarified by testing four derivatives of the bromo compound **5g** bearing benzoyl residues with various substituents, the quinazolinone amide **7**–**10**, previously prepared [12] (shown in Figure 4).

### 2.2. Impact of Quinazolinones on HeLa/NF-kB Luc Cell Survival

**Figure 5:**
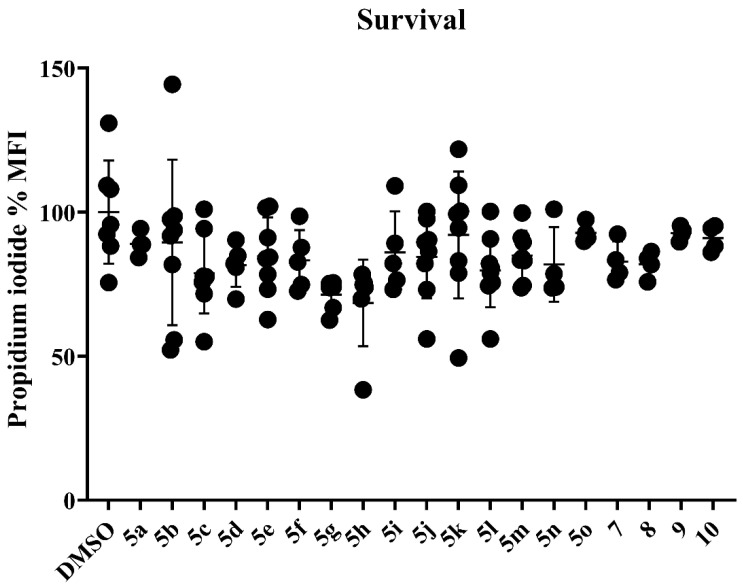
Impact of synthesised quinazolinones of HeLa/NF-κB Luc reporter cell viability. Cells were incubated for 24 h in the absence or presence of 10 μM of each compound, or vehicle control (DMSO at 1:10,000 dilution), followed by propidium iodide staining and detection by flow cytometry. Data represents mean ± SD (N = 4–8). One-way ANOVA was performed, and no signficant differences were observed. DMSO, dimethyl sulfoxide; % MFI, percentage mean fluorescence intensity.

We initially examined whether the compounds had adverse side effects, such as reducing viability/survival of the HeLa/NF-kB Luc cells. For this, cell cultures were incubated in the absence or presence of 10 μM of each compound for 24 h, followed by propidium iodide viability staining, as described under Materials and Methods. Control cultures were incubated in the presence of the same dilution of dimethyl sulfoxide (DMSO). As shown in Figure 5, none of the compounds had any significant impact on cell viability for the duration of the incubation.

### 2.3. Specific Quinazonolinones Inhibit NF-κB Activation in TNF-Induced HeLa/NF-κB-Luc Cells

**Figure 6:**
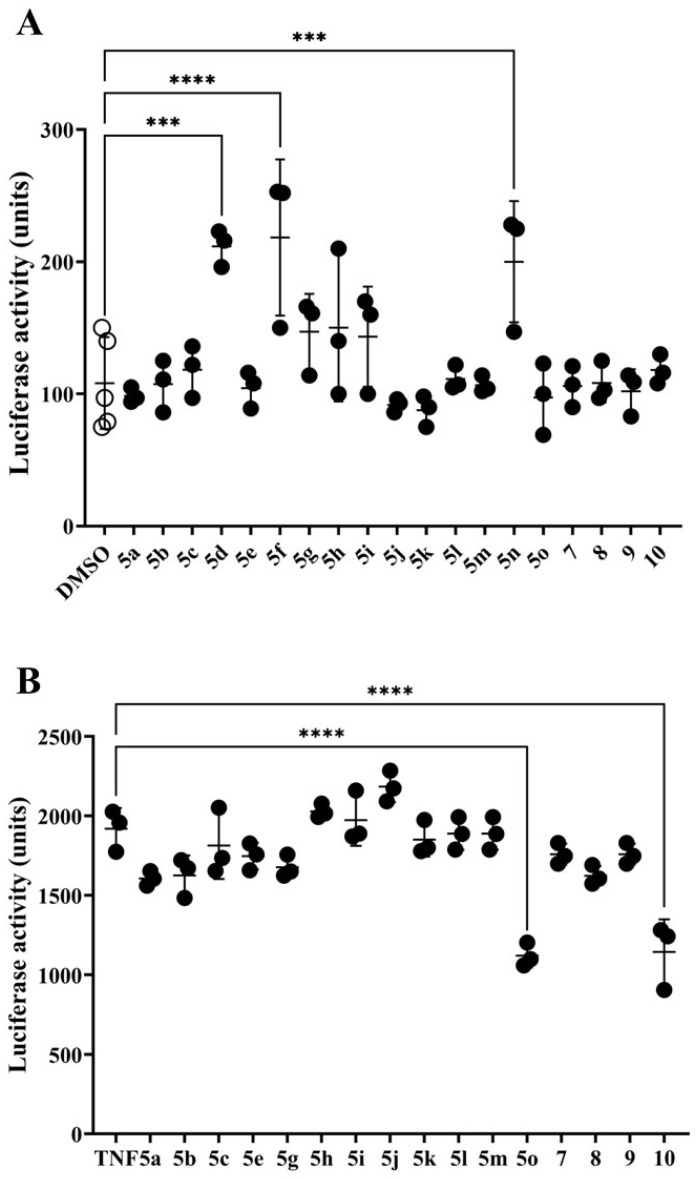
Impact of compounds on NF-κB-regulated luciferase activity in HeLa/NF-κB-Luc cells. (**A**) Cultures were pre-incubated with 10 μM of each QNZ compound and were either left unstimulated for 3 h, or (**B**) were stimulated with 20 ng/mL TNF for 3 h. At the end of experiment the luciferase activity was measured. Data are expressed as mean ± SD, and representative of N = 3 independent experiments (where replicates were n = 5 for DMSO controls, and n = 3 for TNF and QNZs). One-way ANOVA was performed, followed by Tukey’s post hoc multiple pairwise comparisons of treatment means. Significant differences observed, *** *p* < 0.001 and **** *p* < 0.0001.

To test the effect of each of the synthesized QNZ compounds on the HeLa/NF-κB-Luc reporter assay, cell cultures were pre-incubated in the presence of each compound alone, at 10 µM for 30 min, followed by a 3 h incubation and luciferase measured as previously reported [36]. The final concentration of the QNZ compounds was selected as 10 μM, a 1:10,000 dilution of the stock solution (100 mM in DMSO), based on previous experiments that established that DMSO at this dilution did not have any impact on the luciferase assay outcome [36]. Among the QNZ compounds tested alone, only **5d**, **5f**, and **5n** had a significant impact on the luciferase assay compared to vehicle-treated controls; all three compounds *p* < 0.001, one-way ANOVA (Figure 6A). These three compounds were therefore excluded from subsequent experiments utilizing TNF as the stimulus of the canonical NF-κB signaling. The effect of the rest of the QNZs on NF-κB activation in HeLa/NF-κB-Luc cell cultures was examined after preincubation for 30 min with 10 μM compound, followed by treatment with 20 ng/mL TNF for 3 h. At the end of the stimulation, the cultures were processed for measurement of luciferase activity. Of the QNZ compounds tested, only **5o** and **10** demonstrated statistically significant inhibition of TNF-induced NF-κB-regulated luciferase expression, by approximately 50% compared to vehicle-pre-treated cells treated with TNF (both compounds *p* < 0.001; one-way ANOVA); see Figure 6B.

**Figure 7:**
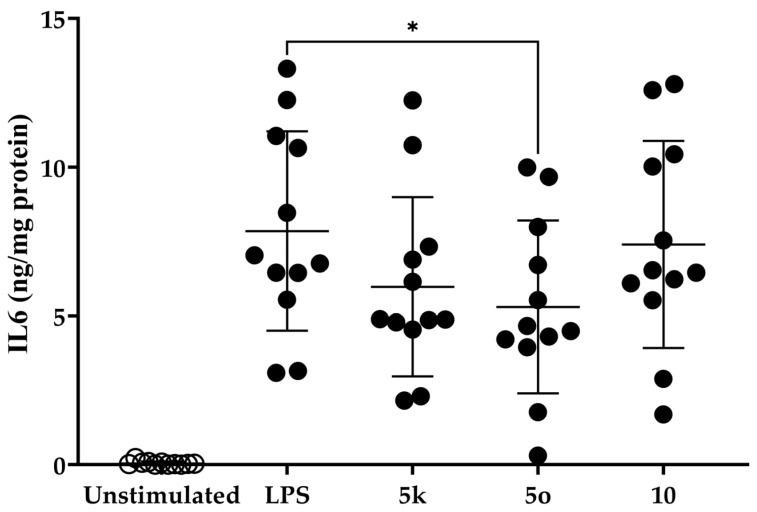
QNZ compound **5o** inhibits production of IL-6 from in vitro cultured human primary macrophages. Peripheral blood mononuclear cell (PBMC)-derived macrophages from 6 healthy donors, were cultured in duplicates and incubated in the absence or presence of 10 μM of specific QNZ compounds (**5k**, **5o** and **10**) for 30 min. Cultures were then stimulated with 100 ng/mL LPS alone or in the presence of QNZ compunds for 24 h (black closed circles). Unstimulated PBMC-derived macrophages (open circles) and LPS alone received DMSO vehicle (1:10,000 dilution). IL-6 levels were detected in the culture medium. Comparisons of treatment means vs. LPS alone, signifcant differences shown * *p* < 0.05 (Mann–Whitney U test, corrected for number of comparisons made).

Given IL-6 is a known target of the canonical NF-κB pathway, the biological activity of QNZ compounds **5o** and **10** observed in relation to NF-κB activation in the luciferase reporter assay was further tested for ability to attenuate LPS-induced IL-6 production by human primary macrophages. QNZ compound, **5k**, that was seen to be ineffective in blocking TNF-induced NF-κB activation in the luciferase assay was also included as a negative control (Figure 6B). Peripheral blood mononuclear cell (PBMC)-derived macrophage cultures from healthy donors were each incubated in the presence of 10 μM of the three selected QNZ compounds (**5k**, **5o**, and **10**) for 30 min and then stimulated with 100 ng/mL of LPS for 24 h. The culture medium was harvested for quantification of IL-6 by ELISA. LPS significantly induced production of IL-6 from human primary macrophage cultures compared to unstimulated controls (*p* < 0.0001); see Figure 7. Among the synthesized QNZ compounds tested, only **5o** significantly attenuated LPS-induced production of IL-6 from macrophages (7.4006 ± 3.3322 ng IL-6/mg protein) compared to vehicle pre-treated, LPS-stimulated controls (7.8539 ± 3.2079 ng IL-6/mg protein); *p* < 0.05 (Figure 7).

### 2.4. Molecular Docking Analysis

To test the possibility that the identified bioactive QNZ compounds exert their effects by directly interfering with NF-κB itself or the whole protein–DNA complex, we proceeded with molecular docking studies to examine the plausibility of this hypothesis and to potentially reveal a mechanism that explains their observed actions in vitro. Molecular docking calculates the binding energy (ΔG) of a ligand to its target. The more negative the binding energy, the stronger the binding. We therefore analyzed possible interactions for active QNZ compounds **5o** and **10**. The top ten binding energies obtained from the docking experiments, indicating the strongest molecular interactions, are shown in Table 2. Both **5o** and **10** showed good binding energies, with lower values for the whole p65/NF-κB-DNA complex compared to p65/NF-κB alone.

## 3. Discussion

In this study, we aimed to synthesize 3-amino-2-methylquinazolin-4(3*H*)one molecules with potential anti-inflammatory activity, using a two-step protocol that was a modification of a previous protocol where microwave irradiation had been utilized [12]. In that respect, an autoclave and a different solvent for the second step were used, as compared to the previous microwave-assisted procedure. In addition, a new one-pot multicomponent reaction was also successfully established. The synthesis of benzoxazinones (**3**) in an autoclave was superior to the thermal one [11] and comparable to the one under microwave irradiation conditions, based, at least, on the reaction time. The yield of the final compounds **5a**–**o** generated by the two-step procedure in the autoclave seemed to give much better results for most of the products. Nevertheless, for the autoclave method, the reaction time should be doubled and the ethanol should be replaced by AcOH, compared to the previous microwave irradiation protocol [12]. Doubling the time for the second step, while increasing the total yield, seems to keep the efficiency of the autoclave procedure comparatively high.

The fact that our team has proposed an operative one-pot synthesis of 3-OH-2-methylquinazolinones [11] has prompted us to attempt a reaction for the one-pot synthesis of 3-NH_2_-2-methylquinazolinones (**5**), simply by exchanging NH_2_OH to NH_2_NH_2_, respectively. Attempting representative examples, it seems that triethyl orthoacetate (CH_3_C(OEt)_3_, **6**) replaced sufficiently Ac_2_O (**2**) in the reaction. In addition, although benzoxazinones (**3**) were used in the second step without any further purification, avoiding their isolation—which is associated with inevitable loss due to handling—has increased the yield of the desired products **5** from 4 to 25%, with **5b** exhibiting the best improvement in yield. As for compounds **5k** and **5l**, the existence of the high electron-withdrawing effect of NO_2_ group at a *p*-position to the NH_2_ of the anthranilic acids did not allow the reaction to proceed to the synthesis of quinazolinones, as has been observed also for the related 3-OH quinazolinones. Nevertheless, the two-step reaction was proved quite efficient.

The biological activity of the new compounds was tested on their ability to affect the activation of the canonical NF-κB pathway using a commercial HeLa/NF-kB Luc reporter cell line. Among the QNZ compounds tested, **5d**, **5f**, and **5n** were observed to induce a luminescence signal, and, given this action, they were omitted from further investigations examining QNZ impact on ligand (TNF)-induced HeLa/NF-kB Luc cell activation. If one considers structural and electronic effects, it seems at first glance that occupation of position 7 of the QNZ scaffold is responsible for the QNZ-induced luminescence response. Nevertheless, derivatives **5h** and **5l** have their substituents (Br) and (NO_2_) also attached to position 7 of the nucleus and they did not show this interference in the luminescence assay. Br is less electronegative than both F and Cl, but significantly bulkier (larger atomic radius). This bulking effect might be an explanation for the observed differences in activity of **5h** compared to **5d** and **5f**, despite all bearing a halogen at position 7. On the other hand, if electronegativity was the only phenomenon of consideration, compound **5l** (7-NO_2_) should be on the list of compounds that induce a luminescence signal in the absence of ligand-induced cell activation, and **5n** (7-OMe) should not, as NO_2_ exhibits a strong –R and OMe a strong +R conjugation effect, respectively. Based on the above points, it is to be concluded that strong electron-withdrawing effects (-I, -R of the nitro group) do not induce luminescence without cell activation, and likewise, bulkiness of the substituent (Br) in position 7.

Among all the quinazolinones tested, only the **5o** (3-amino-6-hydroxy-2-methylquinazolin-4(3*H*)-one) and **10** (N-(6-bromo-2-methyl-4-oxoquinazolin-3(4*H*)-yl)-4-nitrobenzamide) demonstrated significant attenuation of TNF-induced NF-κB-regulated luciferase expression, with **5o** showing further biological importance through its ability to reduce production of pro-inflammatory cytokine IL-6 from human primary macrophage cultures. As far as NF-κB-regulated luciferase activity is concerned, a structure activity relationship cannot be attempted between **5o** and **10**, because **5o** is compared with all derivatives bearing substituents on the aromatic nucleus of QNZ (positions 5–8), while keeping the amino group intact on the right part of the molecule. Importantly, a quinazolinone bearing OH at R^1^ (6 position), such as compound **5o**, confers blockade of NF-κB activity. It seems that nitro groups of derivatives **5l, 5m** with the strong –I and –R withdrawing inductive and resonance effects, might be the reason for the reduced activity. As F, Cl, Br, I, OMe, and OH substituents (in QNZ compounds **5c**, **5e**, **5g**, **5j**, **5m** and **5o**, respectively) induce (with differentiations) all –I inductive and +R resonance effects, only **5o** is active. That means that the hydrogen bond capacity of **5o** is crucial for the inhibitory effect. In addition, as **5o** is very closely related to **5m** (OH versus OMe, respectively) the conclusion of the necessity of hydrogen bonding is strengthened. This type of interaction has been reported previously in relation to NF-κB inhibition by green tea catechins [37] and curcumin [38]. Synthesized QNZ compound **10**, however, probably opens new roads for the substitution on the 3-amino group. It is obvious that insertion of the *p*-NO_2_-Ph group to **5g** transforms the non-active **5g** derivative into an active one (**10**). It might be concluded that derivatization of the most potent QNZ compound **5o** towards amidation of position 3 is worthy, and this will be our future attempt. This is also supported by molecular docking studies, which show a high increase in the binding capacity of QNZ compound **10** to the NF-κB-DNA complex. Our study has identified a potential inhibitor of the canonical NF-κB pathway. Nevertheless, the biological assays presented here that demonstrate this promising activity are limited and future studies utilizing alternative cellular or whole animal models, as well as direct biochemical and molecular experiments, to provide complementary mechanistic insight will be beneficial and substantially increase the impact of this work.

## 4. Materials and Methods

### 4.1. Quinazolinones

The novel quinazolinones were synthesized as part of this study in the Laboratory of Organic Chemistry, Department of Chemistry, Aristotle University of Thessaloniki, Greece. All compound stock solutions were solubilized in dimethyl sulfoxide (DMSO) (Sigma-Aldrich, Poole, UK) to a final concentration of 100 mM and stored at room temperature.

#### 4.1.1. General Methods

The reagents used in this study were anthranilic acids **1a**–**o** (Fluorochem Ltd.; Hadfield, UK), NH_2_NH_2_ (Merck; Darmstadt, Germany), AcOH (Fluka/Fisher Scientific; Loughborough, UK), and CH_3_C(OEt)_3_ (ThermoFisher Scientific; Waltham, MA, USA). The nuclear magnetic resonance (NMR) spectra were recorded on an Agilent 500/54 (500 MHz and 125 MHz for ^1^H and ^13^C, respectively) spectrometer (Agilent Technologies; Santa Clara, CA, USA) using deuterated chloroform (CDCl_3_) and/or hexadeuterodimethyl sulfoxide (DMSO-d_6_) as solvent (Fluorochem Ltd.). J values are reported in Hz. Microwave irradiation experiments were performed on a scientific focused microwave reactor (Biotage Initiator 2.0—Biotage AB; Uppsala, Sweden). All reactions were monitored on commercially available pre-coated thin layer chromatography (TLC) plates (layer thickness of 0.25 mm; Kieselgel 60 F254—Merck; Darmstadt, Germany). The calculation of the yields was based on the amount of the directly crystallized product or after purification (when needed) and recrystallization. The melting points were measured with a Gallenkamp MFB-595 melting point apparatus (Gallenkamp; Cambridge, UK) and are uncorrected. All 3-amino-2-methylquinazolinones **5a**–**o** were known and their data were compared with the ones given in the literature.

#### 4.1.2. Two-Step Synthesis of 3-Amino-2-methylquinazolinones

Step A. Synthesis of benzoxazinones **3a**–**o**: Commercially available anthranilic acids (1 mmol) **1a**–**o** reacted in Ac_2_O **2** (0.5 M) in an autoclave at 120 °C for 20 min to give the corresponding benzoxazinones **3a**–**o** which were obtained upon cooling from the reaction mixture by filtration. The filtrate was washed with petroleum ether and dried under vacuum.

Step B. Synthesis of quinazolinones **5a**–**o**: Benzoxazinones **3a**–**o** were then dissolved in AcOH (0.5–1 mL) where NH_2_NH_2_·H_2_O (**4**) (2 mol equiv.) was added and the mixture was heated in an autoclave, at 150 °C for 30–60 min. Water was added (1 mL) to increase product precipitation. The solid formation was filtrated, washed with water, dried, and recrystallized from the proper solvent. Column chromatography was needed only in case of nitro derivatives **5k**,**l**.

#### 4.1.3. One-Pot Synthesis of 3-Amino-2-methylquinazolinones

General Procedure: Anthranilic acids **1** were individually dissolved in AcOH (0.5 mL) and CH_3_C(OEt)_3_ (**6**) (2 mol equiv.), and NH_2_NH_2_·H_2_O (**4**) (2 mol equiv.) were added. The mixtures were microwave irradiated at 150 °C for 60–90 min. Upon cooling of the reaction mixture, water was added (1 mL) and the precipitate formed was filtrated, washed with water, and dried under vacuum. Recrystallization provided the purified product.

### 4.2. Data of Compounds

^1^H and ^13^C-NMR spectra of compounds **5d**, **5h**, **5m,** and **5n** are provided in Supporting Information. ^1^H NMR spectra are provided for all compounds synthesized with the two-step new method. When no reference is provided, data of compounds were compared to those provided in [12].

3-amino-2-methylquinazolin-4(3H)-one (**5a**): One-pot MW reaction time (rt): 75 min; Two-Step Synthesis, Step B rt: 30 min: ^1^H-NMR (CDCl_3_, 500 MHz): *δ* (ppm) 8.19 (d, *J* = 7.6 Hz, 1H), 7.70 (t, *J* = 7.1 Hz, 1H), 7.61 (d, *J* = 7.7 Hz, 1H), 7.42 (t, *J* = 7.1 Hz, 1H) 4.92 (s, 2H), 2.69 (s, 3H).

3-amino-2,6-dimethylquinazolin-4(3H)-one (**5b**): One-pot MW rt: 75 min; Two-Step Synthesis, Step B rt: 60 min: ^1^H-NMR (DMSO-*d6*, 500 MHz): *δ* (ppm) 7.89 (s, 1H, H_5_), 7.61 (dd, *J* = 7.8, 1.8 Hz, 1H, H_7_), 7.50 (d, *J* = 7.8 Hz, 1H, H_8_), 5.70 (very broad singlet, 2H, NH_2_), 2.56 (s, 3H, CH_3_), 2.44 (s, 3H, CH_3_).

3-amino-6-fluoro-2-methylquinazolin-4(3H)-one (**5c**): One-pot MW rt: 75 min; Two-Step Synthesis, Step B rt: 45 min: ^1^H-NMR (DMSO-*d6*, 500 MHz): *δ* (ppm) 7.76 (d, *J* = 8.1 Hz, 1H, H_7_), 7.68–7.63 (m, 2H, H*_5_*, H_8_), 5.82 (s, 2H, NH*_2_*), 2.57 (s, 3H, CH*_3_*).

3-amino-7-fluoro-2-methylquinazolin-4(3H)-one (**5d**) [39]: Two-Step Synthesis, Step B rt: 45 min. ^1^H-NMR (CDCl_3_, 300 MHz): *δ* (ppm) 8.21 (dd, ^3^*J_HH_* = 8.9, ^4^*J_HF_* = 6.1 Hz, 1H, H_5_), 7.26 (dd, ^3^*J_HF_* = 9.8 Hz, ^4^*J_HH_* = 2.5 Hz, 1H, H_8_), 7.14 (ddd, ^3^*J_HF_* = 8.4, ^3^*J_HH_* = 8.4, ^4^*J_HH_* = 2.5 Hz, 1H, H_6_), 4.87 (s, 2H, NH*_2_*), 2.69 (s, 3H, CH*_3_*); ^13^C NMR (CDCl_3_, 75 MHz): *δ* (ppm) 166.4 (d, ^1^*J_CF_* = 252.3 Hz), 160.9, 156.8, 149.0 (d, ^3^*J_CF_* = 13.1 Hz), 129.2 (d, ^3^*J_CF_* = 10.7 Hz), 116.8, 115.3 (d, ^2^*J_CF_* = 23.5 Hz), 112.2 (d, ^2^*J_CF_* = 21.9 Hz), 22.3.

3-amino-6-chloro-2-methylquinazolin-4(3H)-one (**5e**): One-pot MW rt: 75 min; Two-Step Synthesis, Step B rt: 45 min. ^1^H-NMR (DMSO-*d6*, 300 MHz): *δ* (ppm) 8.01 (s, 1H, H_5_), 7.78 (dd, *J* = 8.6, 2.2 Hz, 1H, H_7_), 7.60 (d, *J* = 8.70 Hz, 1H, H_8_), 5.83 (s, 2H, NH_2_), 2.57 (s, 3H, CH_3_).

3-amino-7-chloro-2-methylquinazolin-4(3H)-one (**5f**): One-pot MW rt: 75 min; Two-Step Synthesis, Step B rt: 60 min: ^1^H-NMR (DMSO-*d6*, 300 MHz): *δ* (ppm) 8.07 (d, *J* = 8.55 Hz, 1H, H_5_), 7.63 (s, 1H, H_8_), 7.49 (dd, *J* = 8.5, 1.4 Hz, 1H, H_6_), 5.80 (s, 2H, NH_2_), 2.57 (s, 3H, CH_3_).

3-amino-6-bromo-2-methylquinazolin-4(3H)-one (**5g**): One-pot MW rt: 90 min; Two-Step Synthesis, Step B rt: 60 min. ^1^H-NMR (DMSO-*d6*, 300 MHz): *δ* (ppm) 8.15 (d, *J* = 3.9 Hz, 1H, H_5_), 7.88 (dd, *J* = 8.7 Hz, 2.3 Hz, 1H, H_7_), 7.53 (d, *J* = 8.7, 1H, H_8_), 5.82 (s, 2H, NH_2_), 2.55 (s, 3H, CH_3_).

3-amino-7-bromo-2-methylquinazolin-4(3H)-one (**5h**) [40]: Two-Step Synthesis, Step B rt. ^1^H-NMR (CDCl_3_, 300 MHz): *δ* (ppm) 8.01 (d, *J* = 8.5 Hz, 1H, H_5_), 7.77 (s, 1H, H_8_), 7.50 (d, *J* = 8.5 Hz, 1H, H_6_), 4.89 (s, 2H, NH_2_), 2.68 (s, 3H, CH_3_); ^13^C-NMR (CDCl_3_, 75 MHz): *δ* (ppm) 161.1, 156.8, 147.9, 129.7, 129.0, 127.9, 118.8, 22.3.

3-amino-6,8-dibromo-2-methylquinazolin-4(3H)-one (**5i**): Two-Step Synthesis, Step B rt. ^1^H-NMR (DMSO-*d6*, 300 MHz): *δ* (ppm) 8.27 (s, 1H, H_5_), 8.14 (d, 1H, H_7_), 5.87 (s, 2H, NH_2_), 2.60 (s, 3H, CH_3_).

3-amino-6-iodo-2-methylquinazolin-4(3H)-one (**5j**): One-pot MW rt: 60 min; Two-Step Synthesis, Step B rt: 30 min. ^1^H-NMR (CDCl_3_, 300 MHz): *δ* (ppm) 8.54 (d, *J* = 1.9 Hz, 1H, H_5_), 7.96 (dd, *J* = 8.6 Hz, 2.1 Hz, 1H, H_7_), 7.34 (d, *J* = 8.6 Hz, 1H, H_8_), 4.89 (s, 2H, NH_2_), 2.68 (s, 3H, CH_3_).

3-amino-2-methyl-6-nitroquinazolin-4(3H)-one (**5k**): Two-Step Synthesis, Step B rt: 60 min. ^1^H-NMR (DMSO–*d6*, 300 MHz): *δ* (ppm) 8.74 (d, *J* = 2.6 Hz, 1H, H_5_), 8.46 (dd, *J* = 9.0, 2.6 Hz, 1H, H_7_), 7.73 (d, *J* = 9.0 Hz, 1H, H_8_), 5.91 (s, 2H, NH_2_), 2.62 (s, 3H, CH_3_).

3-amino-2-methyl-7-nitroquinazolin-4(3H)-one (**5l**): Two-Step Synthesis, Step B rt: 60 min. ^1^H-NMR (DMSO-*d6*, 300 MHz): *δ* (ppm) 8.32–8.23 (m, 2H, H_5_, H_8_), 8.14 (d, *J* = 8.6, 1H, H_6_), 5.91 (s, 2H, NH_2_), 2.61 (s, 3H, CH_3_).

3-amino-6-methoxy-2-methylquinazolin-4(3H)-one (**5m**) [41]: Two-Step Synthesis, Step B rt: 45 min. ^1^H-NMR (CDCl_3_, 300 MHz): *δ* (ppm) 7.54 (d, *J* = 6.3 Hz, 1H, H_8_), 7.52 (s, 1H, H_5_), 7.30 (dd, *J* = 8.8, 2.8 Hz, 1H, H_7_), 4.92 (s, 2H, NH_2_), 3.89 (s, 3H, OCH_3_), 2.66 (s, 3H, CH_3_); ^13^C NMR (CDCl_3_, 75 MHz): *δ* (ppm) 161.4, 157.9, 153.0, 141.7, 128.5, 124.8, 120.7, 105.6, 55.8, 22.0.

3-amino-6,8-dimethoxy-2-methylquinazolin-4(3H)-one (**5n**) [42]: Two-Step Synthesis, Step B rt: 45 min. ^1^H-NMR (DMSO-*d6*, 300 MHz): *δ* (ppm) 7.36 (s, 1H, H_5_), 7.04 (s, 1H, H_8_), 5.76 (s, 2H, NH_2_), 3.88 (s, 3H, OCH_3_), 3.86 (s, 3H, OCH_3_), 2.53 (s, 3H, CH_3_); ^13^C-NMR (DMSO, 75 MHz): *δ* (ppm) 159.8, 154.8, 154.1, 148.6, 143.3, 113.2, 107.7, 105.1, 56.3, 56.1, 22.1.

3-amino-6-hydroxy-2-methylquinazolin-4(3H)-one (**5o**): One-pot MW rt: 75 min; Two-Step Synthesis, Step B rt: 60 min. ^1^H-NMR (DMSO-*d6*, 300 MHz): *δ* (ppm) 9.97 (s, 1H, OH), 7.44 (d, *J* = 8.7 Hz, 1H, H_8_), 7.35 (s, 1H, H_5_), 7.21 (d, *J* = 8.6 Hz, 1H, H_7_), 5.73 (s, 2H, NH_2_), 2.50 (s, 3H, CH_3_).

### 4.3. Tissue Culture and Reagents

The in vitro experiments were performed using the human NF-κB luciferase reporter HeLa stable cell-line (HeLa/NF-κB-Luc; SL-0001—Signosis; Santa Clara, CA, USA) that expresses inducible luciferase under the promoter of the canonical NF-κB pathway. After initial selection over 3 days using 50 μg/mL hygromycin B (#10687010—Invitrogen; Paisley, UK), cultured cells were routinely maintained in Dulbecco’s modified Eagle medium (DMEM), 10% (*v*/*v*) fetal bovine serum (FBS), 1% non-essential amino acids, 10 U/mL penicillin, 10 mg/mL streptomycin and 2 mM L-glutamine, (all Sigma-Aldrich; Poole, UK), in a humidified incubator at 37 °C, in an atmosphere of 5% CO_2_. For passaging, cells were detached upon treatment with 0.05% (*w*/*v*) Trypsin-EDTA solution (Gibco; Paisley, UK) for 5 min at 37 °C and counted using a Neubauer cell-counting chamber (Sigma-Aldrich). Cells in suspension were then re-seeded to flasks, either for maintenance or to plates for experimentation. Human recombinant tumor necrosis factor (TNF; PeproTech; London, UK) at 20 ng/mL, was used to stimulate the HeLa/NF-κB-Luc cells.

### 4.4. Luciferase Reporter Assay

HeLa/NF-κB-Luc cells were seeded in 96-well plates (Greiner CELLSTAR flat bottom; Sigma-Aldrich, Poole, UK) at 0.5 × 10^4^ cells/well in 100 μL complete DMEM medium and incubated overnight (37 °C, 5% CO_2_). Cells were pre-treated with QNZ compounds (10 μM) for 30 min, followed by stimulation with TNF (20 ng/mL) for 3 h. Medium was removed, cells were washed with PBS, and Bright-Glo lysis reagent added (E2620—Promega; Southampton, UK). After 5 min incubation at room temperature, lysates were transferred to 96-well white walled plates (Nunc-Immuno microwell P8616; Sigma-Aldrich) and luminescence was measured using an EnSpire multimode plate reader (PerkinElmer; Waltham, MA, USA), as described previously [36].

### 4.5. Viability Assay

HeLa/NF-κB-Luc cells were cultured in 12-well plates (Greiner CELLSTAR; Sigma-Aldrich) at 0.5 × 10^6^ cells/well in 2 mL medium and incubated overnight. The next day, cells were either left untreated, or they were treated with each of the compounds at a final concentration of 10 μM for 24 h. At the end of the treatment, the cells were washed with cold phosphate-buffered saline (PBS) pH 7.3, scraped, and resuspended in 100 μL PBS. Cells were stained with propidium iodide (Invitrogen; Paisley, UK) according to manufacturer guidelines and analyzed by flow cytometry using a Cytomics FC 500 Beckman Coulter flow cytometer (Beckman Coulter, Inc.; Fullerton, CA, USA). Raw data analysis was performed using the CXP software 2.0 (Beckman Coulter).

### 4.6. Human Primary Macrophage Cultures and Interleukin-6 ELISA

Human peripheral blood mononuclear cells (PBMCs) were obtained from healthy volunteer blood taken. Ethical approval for the studies using human peripheral blood- derived mononuclear cells reported here was obtained from the Ethics Committee for Research of University of Thessaly (54/5 June 2025), with all experiments performed under the supervision of Dr. S. Papoutsopoulou. Informed, written consent was obtained from the healthy adult participants. Peripheral venous blood (2 mL) was immediately heparinized (unfractionated heparin sodium, at 5 U/mL; Wockhardt UK Ltd.; Wrexham, UK). Each sample was mixed 1:2 with sterile PBS, layered over 20 mL Ficoll-Paque™ plus (ThermoFisher Scientific) and centrifuged at 400× *g* for 40 min at room temperature. Peripheral blood mononuclear cells (PBMCs) were aspirated, washed with sterile PBS, resuspended in 1 mL of freezing medium [88% *v*/*v* FCS (Sigma-Aldrich; Poole, UK) plus 12% *v*/*v* DMSO], and stored at −80 °C. Frozen isolated PBMCs were thawed and plated (0.5 × 10^6^ cells/well) in 96-well plates (Nunclon Vita surface; ThermoFisher Scientific) in 0.1 mL of differentiation medium (DMEM, 10% *v*/*v* FCS, 10 mM HEPES (Sigma-Aldrich), 1 mM sodium pyruvate (ThermoFisher Scientific), 1x MEM non-essential amino acids (Thermo Fisher Scientific), 10 U/mL penicillin, 10 mg/mL streptomycin, 2 mM L-glutamine (Sigma-Aldrich) and 50 ng/mL human macrophage colony-stimulating factor (Peprotech; London, UK)]. On day one, non-adherent cells were washed, and fresh differentiation medium was added. On day four, 3 mL of fresh medium was added into the cultures which were incubated for a further 3 days. On the day of the experiment, cells were either left untreated or pre-treated with selected compounds (10 μM) for 30 min prior to stimulation. Lipopolysaccharide (LPS) isolated from adherent, invasive *E. coli* LF82 was used for macrophage Toll-like receptor 4 (TLR4) activation at a final concentration of 100 ng/mL, as previously reported [43]. Cell culture medium was harvested and IL-6 quantified using the Invitrogen human IL-6 uncoated ELISA kit (Cat. number 88-706-88) sourced from Life Technologies Ltd. (Paisley, UK). Cells were washed once in PBS and lysed in 50 μL radioimmunoprecipitation assay (RIPA) buffer (Sigma-Aldrich) and the total protein of the cell lysates were measured using the Pierce Bicinchoninic Acid (BCA) kit (ThermoFisher Scientific).

### 4.7. In Silico Molecular Docking

Docking software AutoDock 4.2 [44] was used to perform blind docking analysis of the human NF-κB potential binding sites for QNZ compounds. The experimental procedure was performed as per AutoDock documentation (https://autodock.scripps.edu/documentation/documentation/, accessed on 17 March 2025). To achieve maximum coverage of p65/NF-κB and the p65/NF-κB-DNA complex during blind docking, only the grid box size was altered beyond the default values of the AutoDock software v. 4.2.6 (The Scripps Research Institute; La Jolla, CA, USA). Specifically, the number of points in x, y, and z dimensions were changed from the default value of 40 to the maximum value of 126. Spacing was kept at the default value of 0.375 Å and the grid box center coordinates were also kept at the default values, where x = 40.522, y = 16.84, and z = 36.636. The algorithm used for setting docking parameters was the Lamarckian algorithm. The default output of the algorithm for each ligand was the 10 most stable binding poses as judged by their binding-energy values in kcal/mol.

## 5. Conclusions

This study compellingly demonstrates novel methodologies for the synthesis of 3-amino-2-methylquinazolinones. Among all molecules tested, only the **5o** that was bearing OH at R^1^ (6 position), inhibited NF-kB activation and the synthesis of IL-6. Collectively, our data shows that QNZ **5o** represents a promising lead compound for optimization in future attempts to target the NF-κB pathway.

## Figures and Tables

**Table 1:** Composition of compounds **1**, **3**, and **5** as far as R^1^, R^2^, and R^3^ is concerned. Yields (%) of product **5** from the two-step autoclave procedure, the two-step microwave (MW) irradiation-assisted procedure (as per [12]), and the one-pot MW procedure.

1, 3, 5	R^1^, R^2^, R^3^	Two-Step Autoclave 5 (%)	Two-Step MW 5 (%)	One-Pot MW 5 (%)
**a**	R^1^ = R^2^ = R^3^ = H	52	35	62
**b**	**R^1^ = CH_3_**, R^2^ = R^3^ = H	57	31	83
**c**	**R^1^ = F**, R^2^ = R^3^ = H	66	51	74
**d**	R^1^ = H, **R^2^ = F,** R^3^ = H	64	np	np
**e**	**R^1^ = Cl**, R^2^ = R^3^ = H	71	50	80
**f**	R^1^ = H, **R^2^ = Cl,** R^3^ = H	67	40	77
**g**	**R^1^ = Br**, R^2^ = R^3^ = H	82	70	86
**h**	R^1^ = H, **R^2^ = Br,** R^3^ = H	85	np	np
**i**	**R^1^ = Br**, R^2^ = H, **R^3^ = Br**	89	85	np
**j**	**R^1^ = I**, R^2^ = R^3^ = H	72	63	78
**k**	**R^1^ = NO_2_**, R^2^ = R^3^ = H	85	85	No reaction
**l**	R^1^ = H, **R^2^ = NO_2_,** R^3^ = H	70	48	No reaction
**m**	**R^1^ = OCH_3_**, R^2^ = R^3^ = H	81	np	np
**n**	R^1^= **OCH_3_**, **R^2^ = OCH_3_,** R^3^ = H	83	np	np
**o**	**R^1^ = OH**, R^2^ = R^3^ = H	58	32	73

np: not performed. The bold text indicates the substituent changes from compound **a** as far as R^1^, R^2^, and R^3^ is concerned.

**Table 2:** Binding energies (kcal/mol) of quinazolinones **5o** and **10** with p65/NF-κB and p65/NF-κB-DNA complex.

5o + p65/NF-κB	5o + p65/NF-κB-DNA Complex	10 + p65/NF-κB	10 + p65/NF-κB-DNA Complex
−5.72	−7.27	−7.69	−11.4
−5.71	−7.25	−7.33	−11.35
−5.63	−7.17	−7.3	−11.3
−5.62	−6.63	−7.28	−11.26
−5.54	−6.61	−7.06	−11.07
−5.53	−6.57	−6.99	−10.73
−5.42	−6.48	−6.65	−10.19
−5.29	−6.48	−6.5	−9.81
−4.78	−6.38	−6.09	−9.7
−4.41	−6.37	−5.96	−9.63

## Data Availability

All the data is presented in the manuscript and within the Supplementary Materials.

## Supplementary Materials

The following supporting information can be downloaded at: https://www.mdpi.com/article/10.3390/ijms27146431/s1.

## Abbreviations

The following abbreviations are used in this manuscript:

Ac_2_O	acetic anhydride
AcOH	acetic acid
ANOVA	analysis of variance
Bcl-2	B-cell lymphoma-2
CH_3_C(OEt)_3_	triethyl orthoacetate
COX-2	cyclooxygenase-2
ΔG	binding energy
IAP	inhibitor of apoptosis
DMSO	dimethyl sulfoxide
EDTA	ethylenediaminetetraacetic acid
IL-1	interleukin 1
IL-6	interleukin 6
LPS	lipopolysaccharide
MFI	mean fluorescence intensity
MW	microwave
NF-κB	nuclear factor-kappa B
NMR	nuclear magnetic resonance
OMe	methoxy group (-OCH_3_)
PBMC	peripheral blood mononuclear cell
QNZ	quinazolinone
TLR4	Toll-like receptor 4
TNF	tumor necrosis factor
VEGFR2	vascular endothelial growth factor receptor 2
WRN	Werner syndrome
