# Supplementary material for: Novel 3-Amino-2-methylquinazolinone NF-κB Inhibitors: Synthesis and Potential Anti-Inflammatory Function
Source: Int J Mol Sci. 2026 Jul 20;27(14):6431. doi: 10.3390/ijms27146431 (PMC13411759; doi:10.3390/ijms27146431)
Supplement: Supplementary file 1 [file ijms-27-06431-s001.zip › ijms-4419333-supplementary.pdf]

Novel 3-Amino-2-methylquinazolinone NF-κB Inhibitors: Synthesis and Potential Anti-Inflammatory Function

Chrysoula Mikra [1,†], Vasiliki Petriki [2,†], Evangelos Tsioupros [2], Eleni Kougioumtzian [2], Konstantinos Michail [2], Stella Manta [1], Barry J. Campbell [3], Konstantina C. Fylaktakidou [1,*] and Stamatia Papoutsopoulou [2,*]

[1] Laboratory of Organic Chemistry, Faculty of Chemistry, Aristotle University of Thessaloniki, 54124 Thessaloniki, Greece; chrmikgeo@chem.auth.gr (C.M.); stmanta@chem.auth.gr (S.M.)

[2] Department of Biochemistry and Biotechnology, University of Thessaly, 41500 Larissa, Greece; vpetriki@uth.gr (V.P.); etsioupros@uth.gr (E.T.); elinakoug@yahoo.gr (E.K.); kostantinemix@gmail.com (K.M.)

[3] Department of Infection Biology and Microbiomes, Institute of Infection, Veterinary and Ecological Sciences, University of Liverpool, Liverpool L69 3GE, UK; bjcampbl@liverpool.ac.uk

* Correspondence: kfylakta@chem.auth.gr (K.C.F.); stapapou@uth.gr (S.P.)

† These authors contributed equally to this work.

NMR spectra of compounds **5a-o**

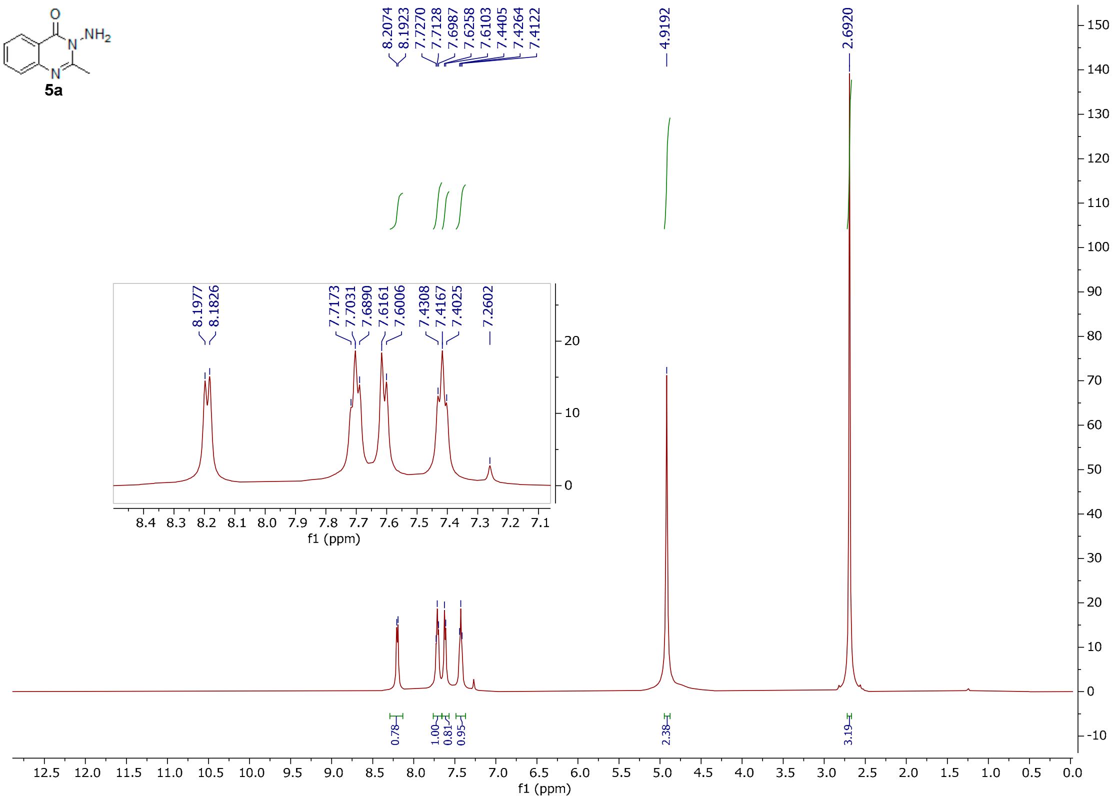

Figure S1: ^{1}H-NMR of 3-amino-2-methylquinazolin-4(3*H*)-one (**5a**)

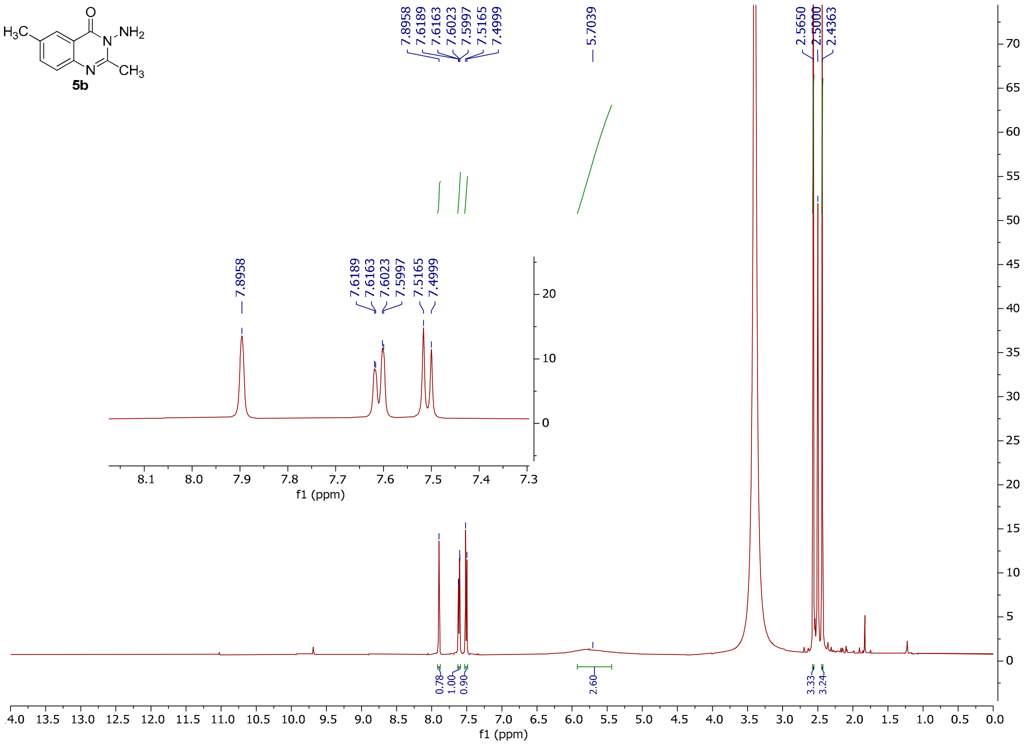

Figure S2: ^{1}H-NMR of 3-amino-2,6-dimethylquinazolin-4(3*H*)-one (**5b**)

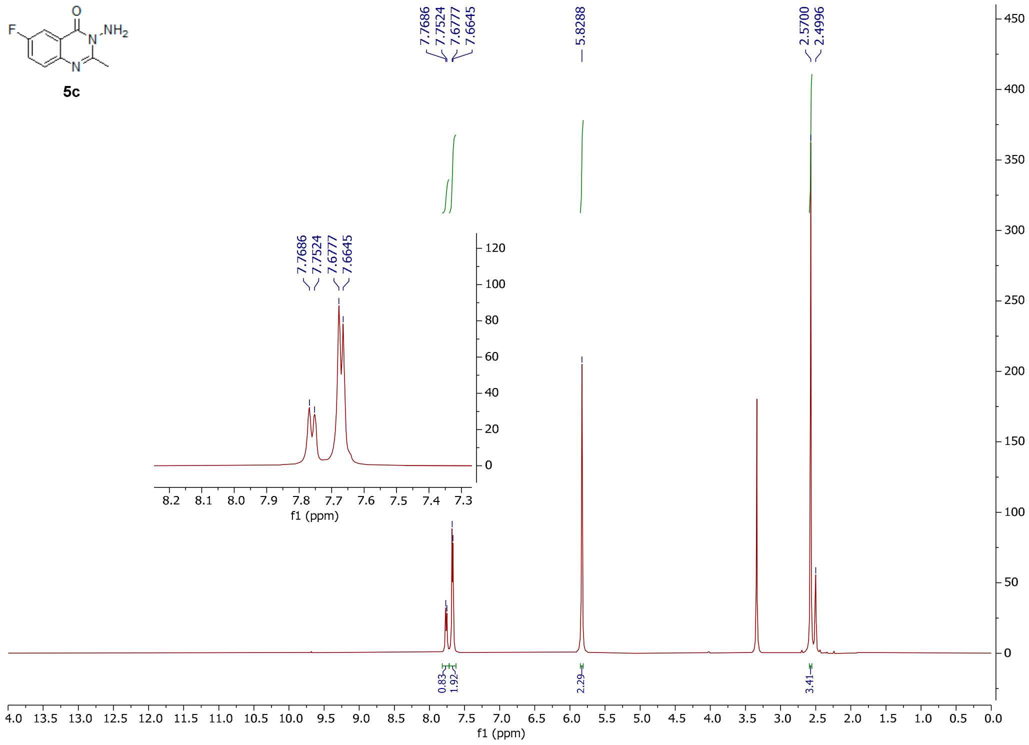

Figure S3: ^{1}H-NMR of 3-amino-6-fluoro-2-methylquinazolin-4(3*H*)-one (**5c**)

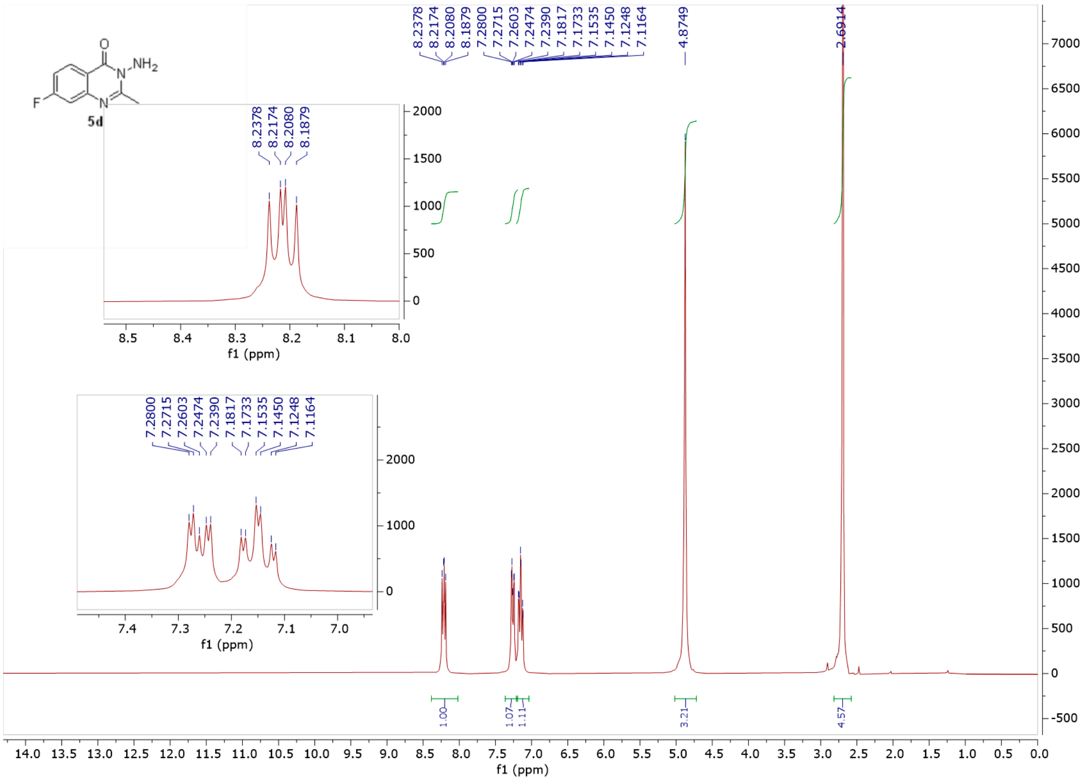

Figure S4: (a) ^{1}H-NMR of 7-fluoro-2-methylquinazolin-4(3*H*)-one (**5d**)

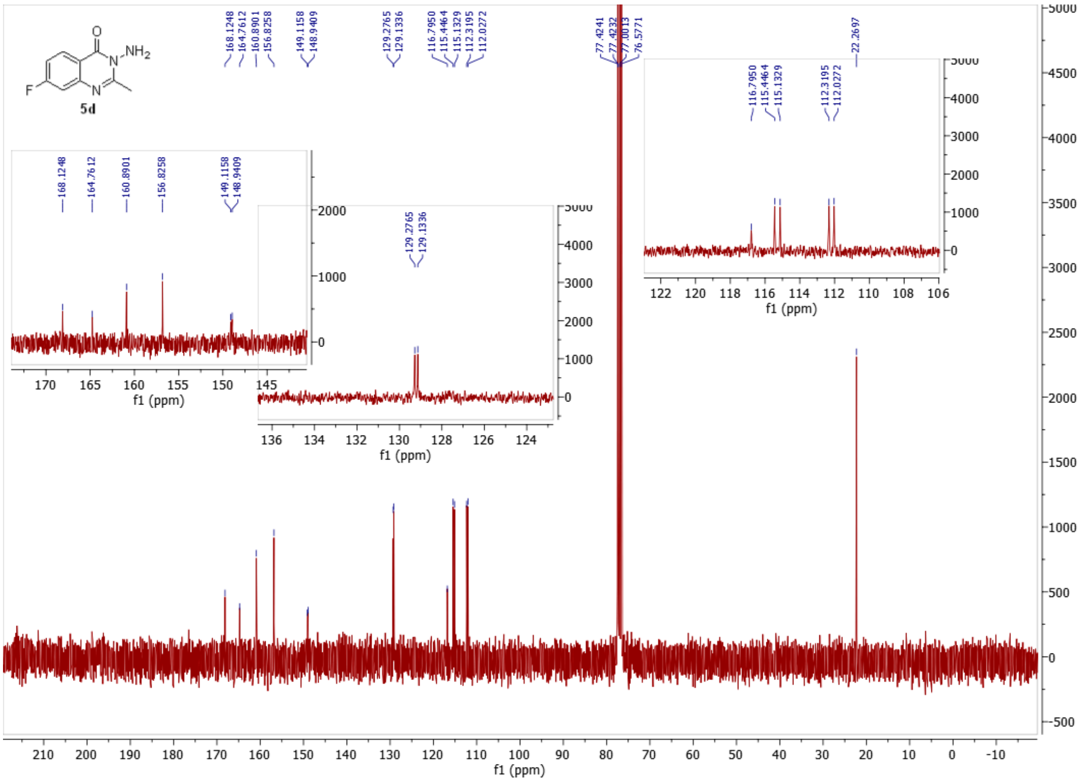

Figure S4: (b) ^{13}C-NMR of 7-fluoro-2-methylquinazolin-4(3*H*)-one (**5d**)

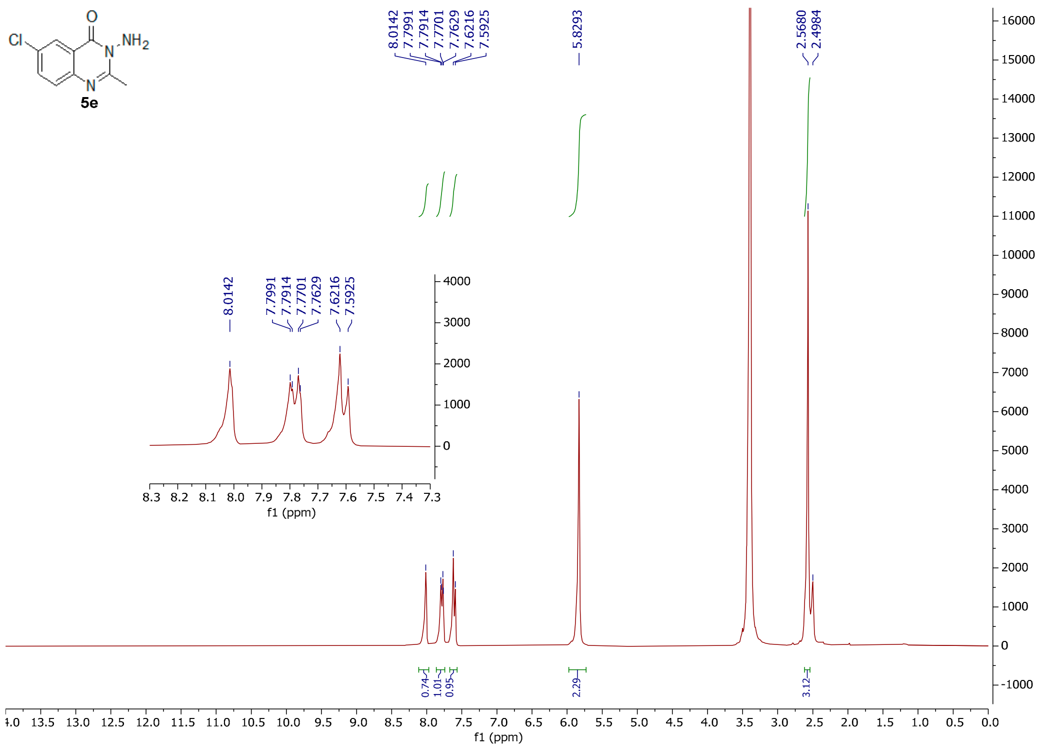

Figure S5: ^{1}H-NMR of 3-amino-6-chloro-2-methylquinazolin-4(3*H*)-one (**5e**)

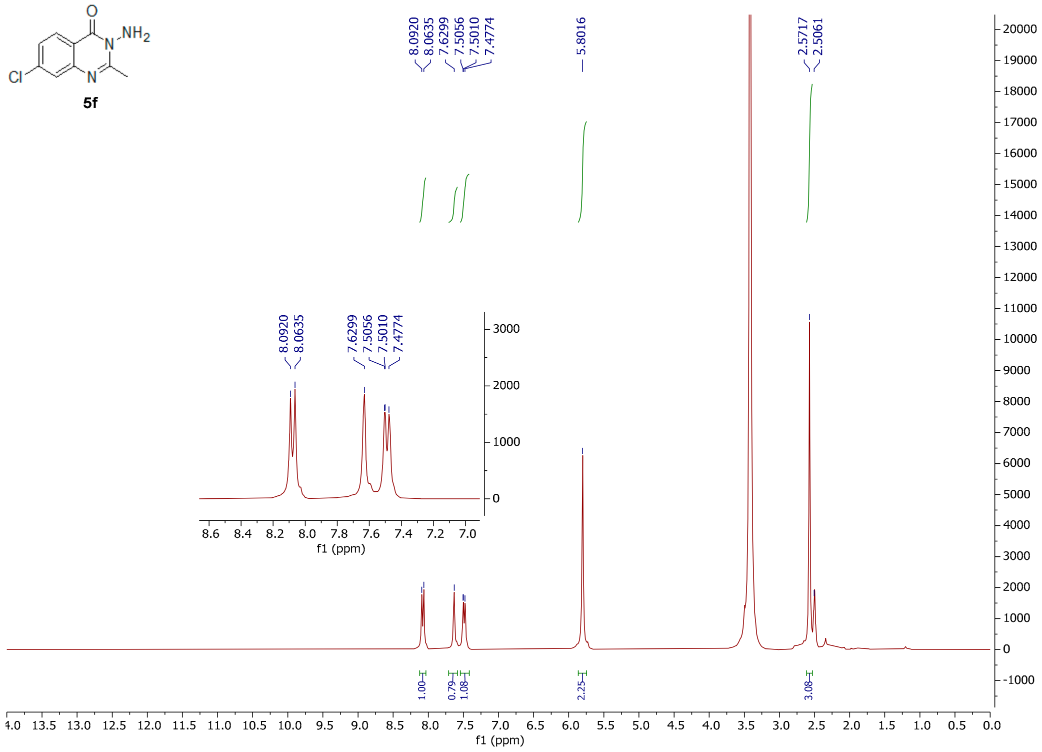

Figure S6: ^{1}H-NMR of 3-amino-7-chloro-2-methylquinazolin-4(3*H*)-one (**5f**)

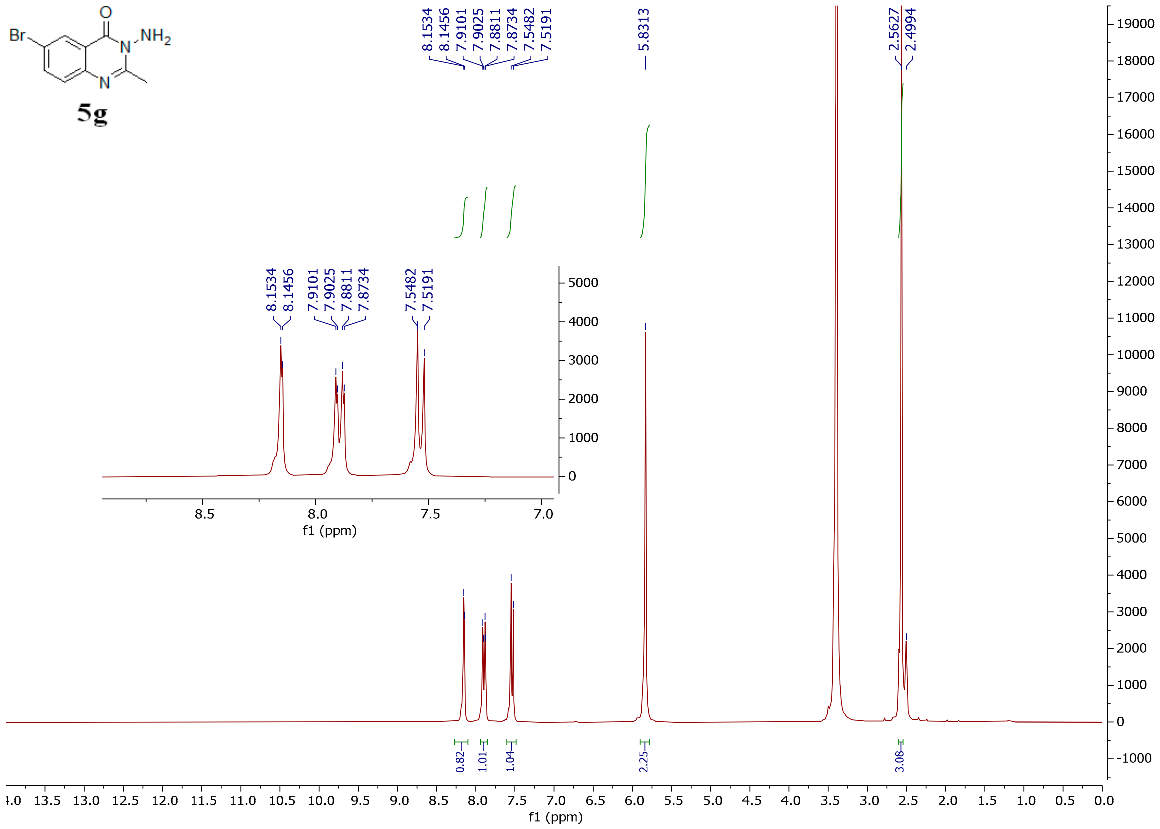

Figure S7: ^{1}H-NMR of 3-amino-6-bromo-2-methylquinazolin-4(3*H*)-one (**5g**)

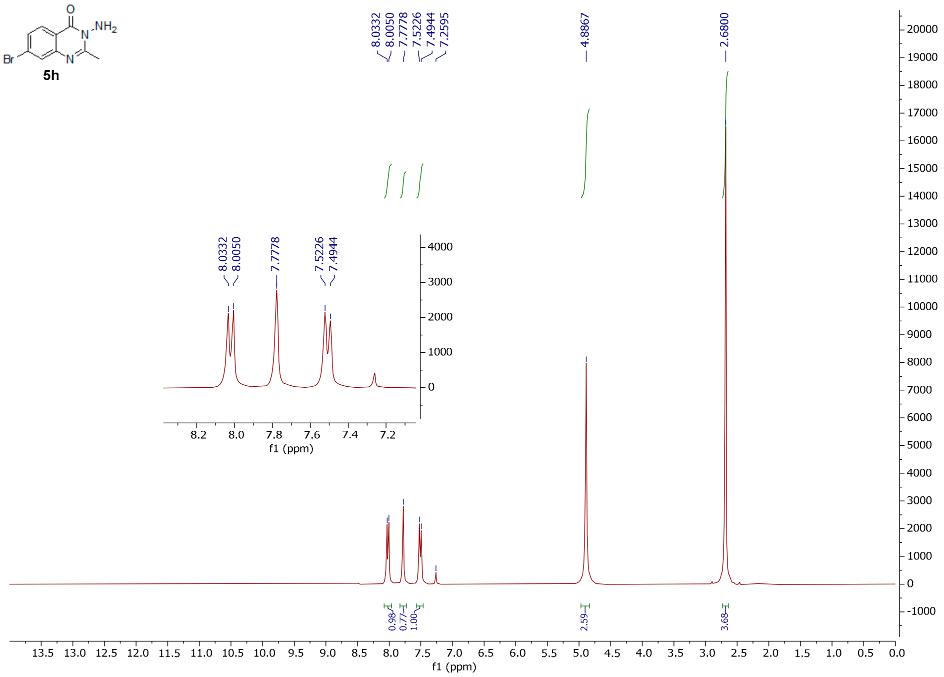

Figure S8: (a) ^{1}H-NMR of 3-amino-7-bromo-2-methylquinazolin-4(3*H*)-one (**5h**)

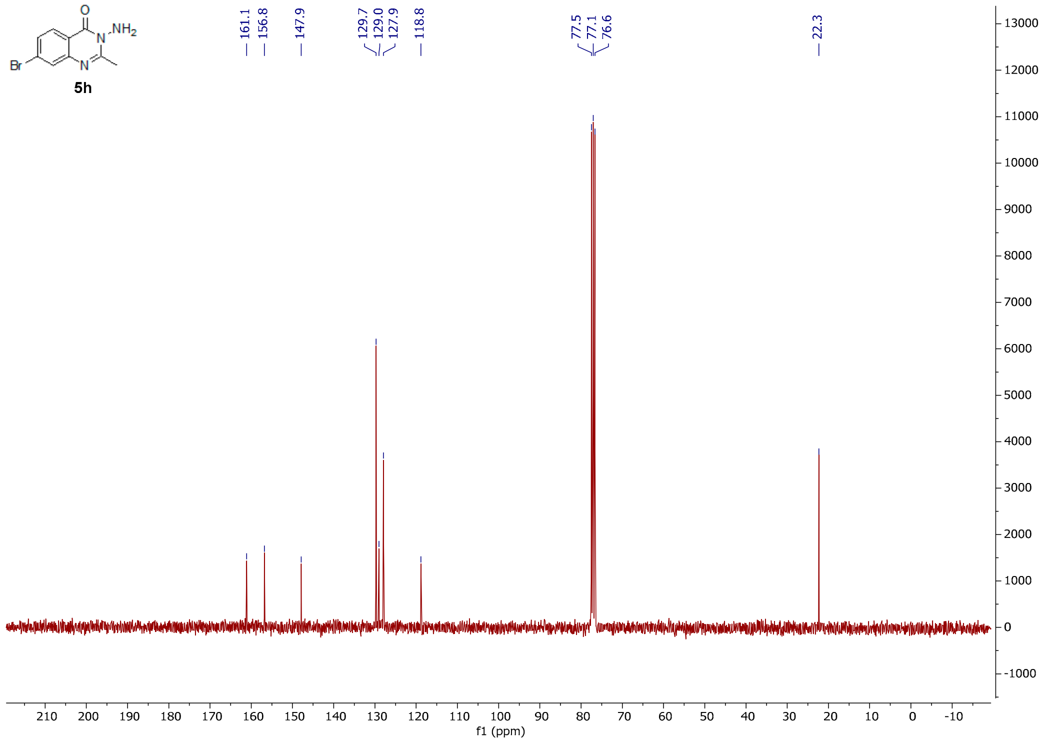

Figure S8: (b) ^{13}C-NMR of 3-amino-7-bromo-2-methylquinazolin-4(3*H*)-one (**5h**)

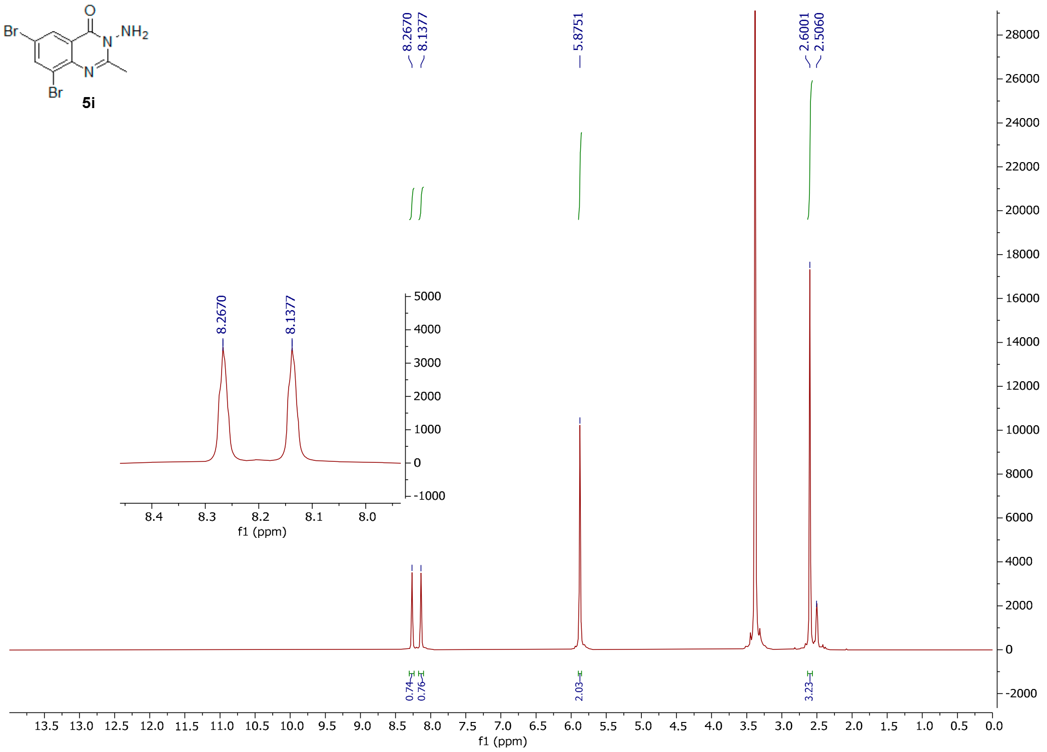

Figure S9: ^{1}H-NMR of 3-amino-6,8-dibromo-2-methylquinazolin-4(3*H*)-one (**5i**)

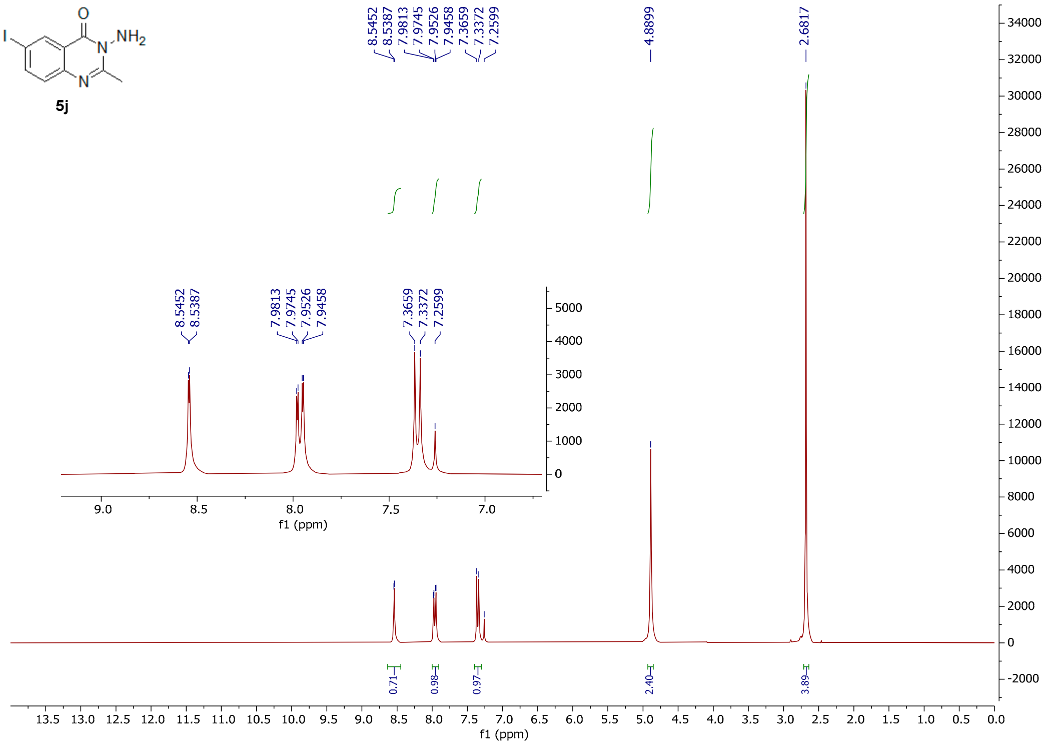

Figure S10: ^{1}H-NMR of 3-amino-6-iodo-2-methylquinazolin-4(3*H*)-one (**5j**)

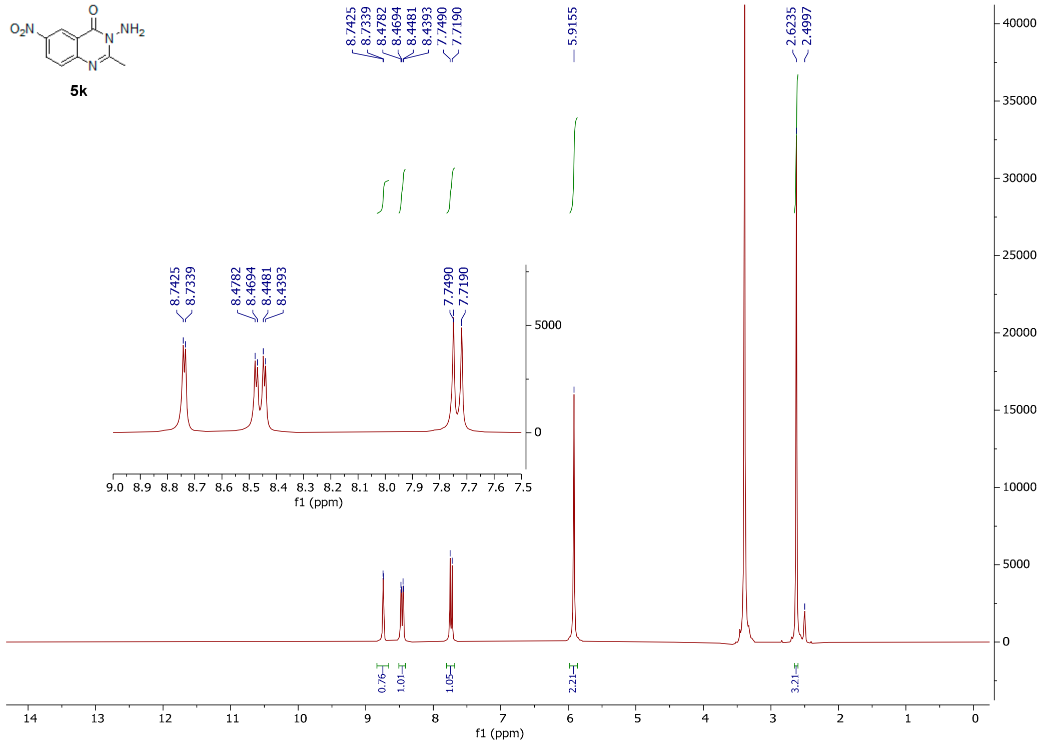

Figure S11: ^{1}H-NMR of 3-amino-2-methyl-6-nitroquinazolin-4(3*H*)-one (**5k**)

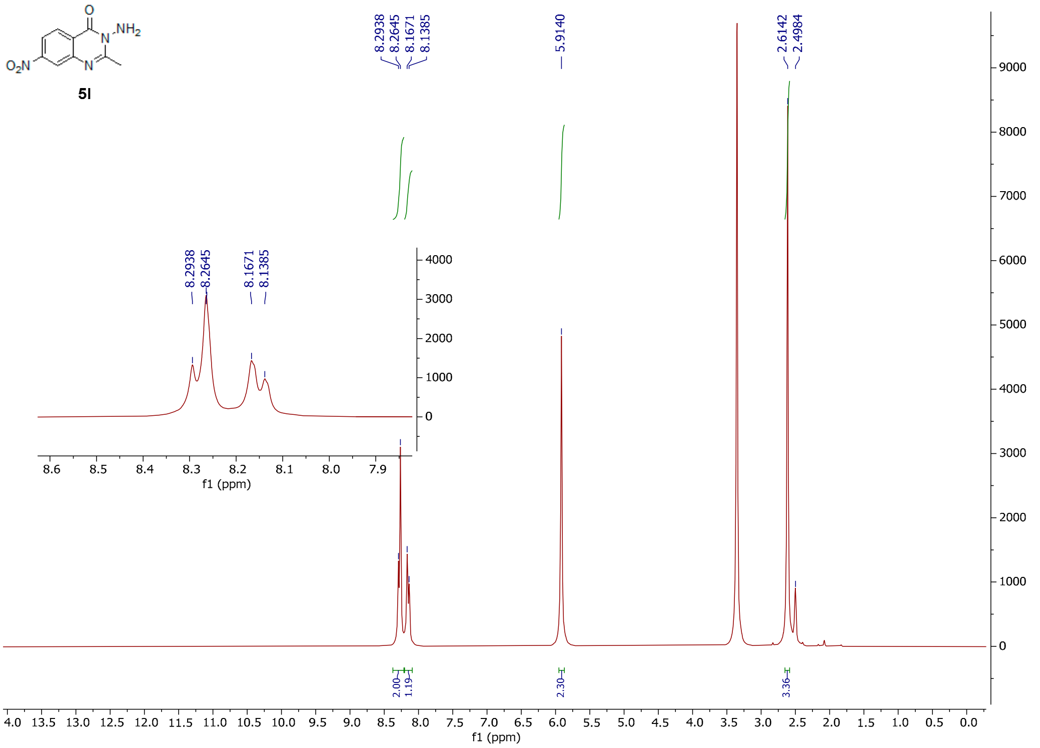

Figure S12: ¹H-NMR of 3-amino-2-methyl-7-nitroquinazolin-4(3*H*)-one (**5l**)

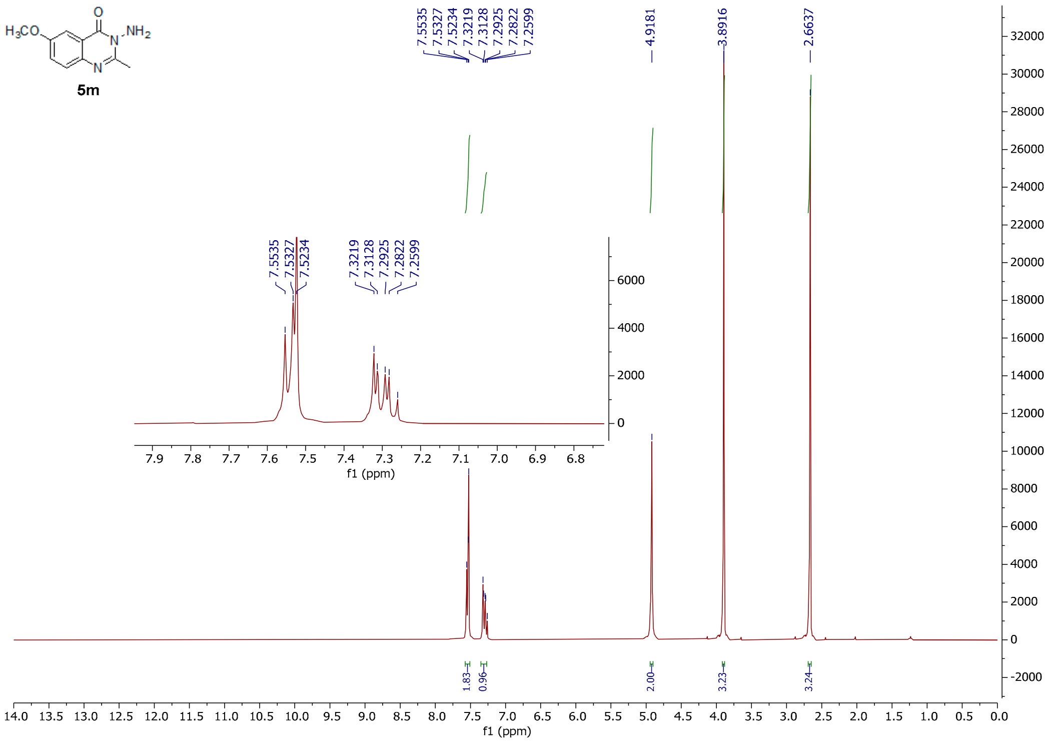

Figure S13: (a) ¹H-NMR of 3-amino-6-methoxy-2-methylquinazolin-4(3*H*)-one (**5m**)

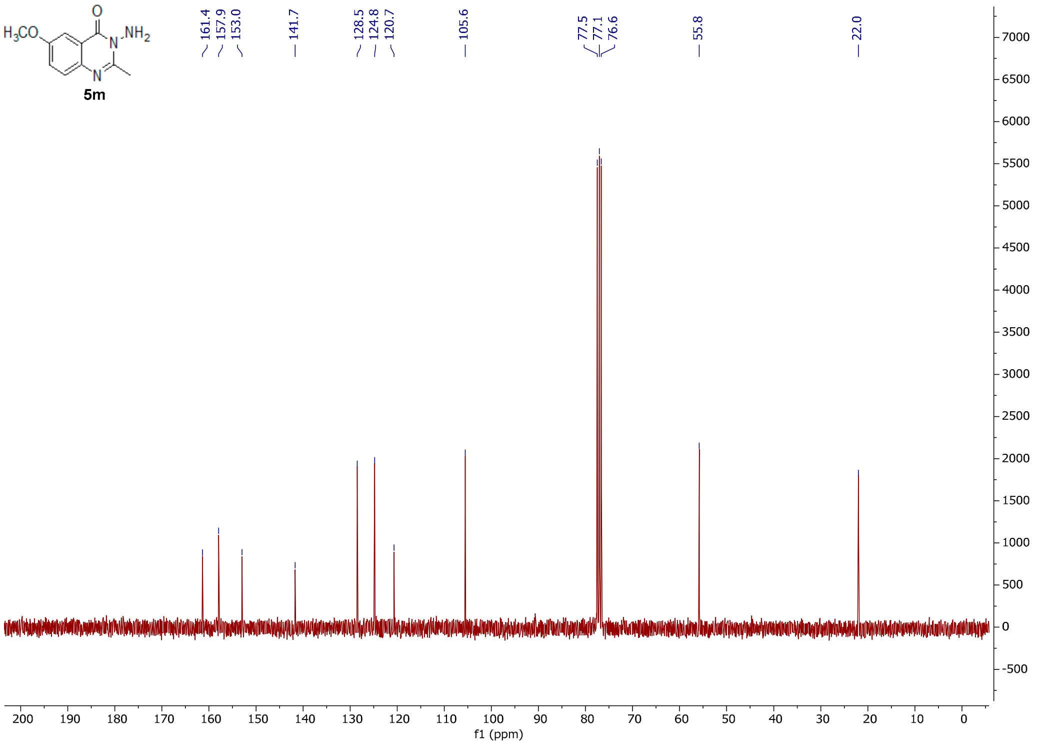

Figure S13: (b) ^{13}C-NMR of 3-amino-6-methoxy-2-methylquinazolin-4(3*H*)-one (**5m**)

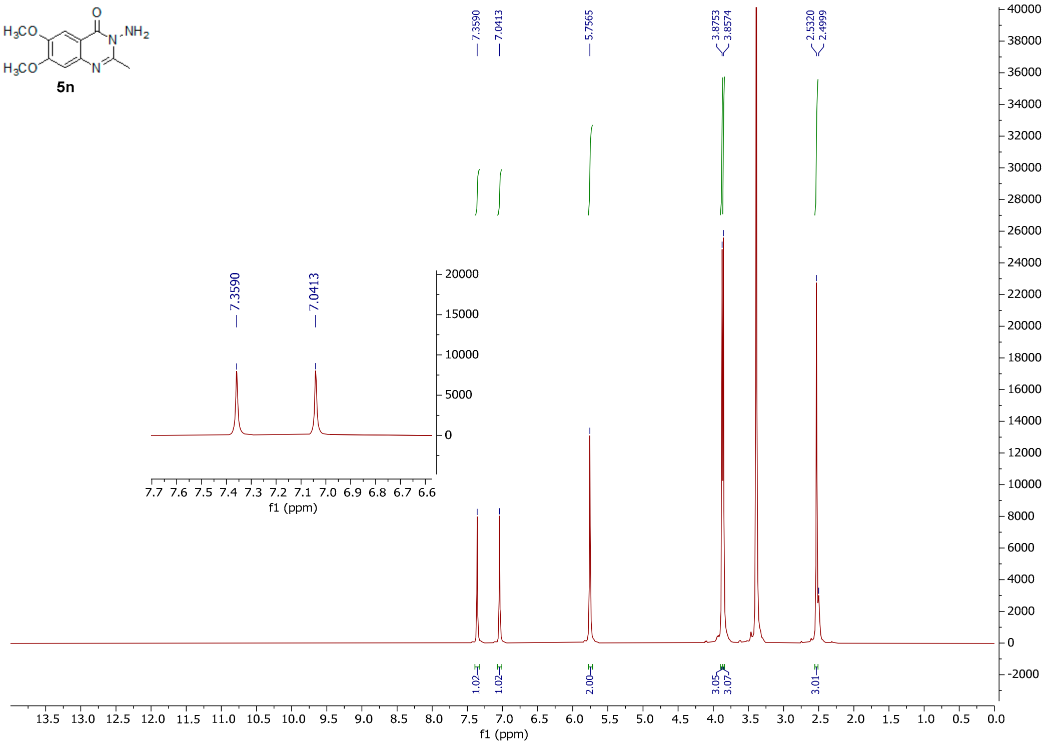

Figure S14: (a) ^{1}H-NMR of 3-amino-6,8-dimethoxy-2-methylquinazolin-4(3*H*)-one (**5n**)

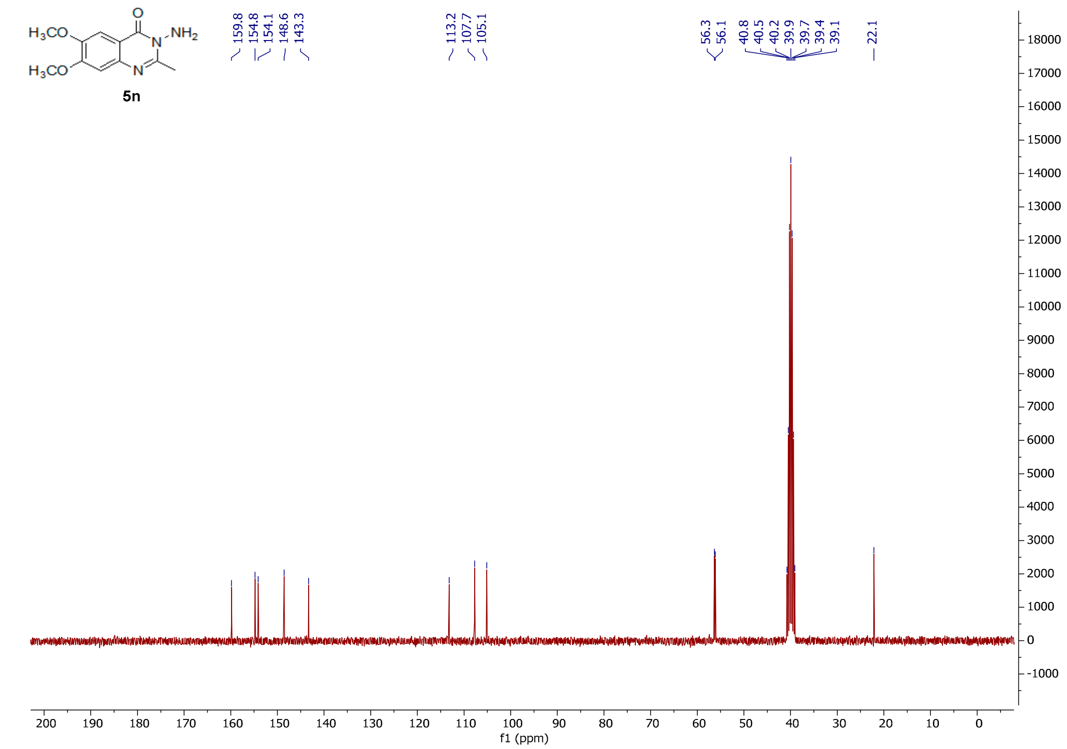

Figure S14: (b) ^{13}C-NMR of 3-amino-6,8-dimethoxy-2-methylquinazolin-4(3*H*)-one (**5n**)

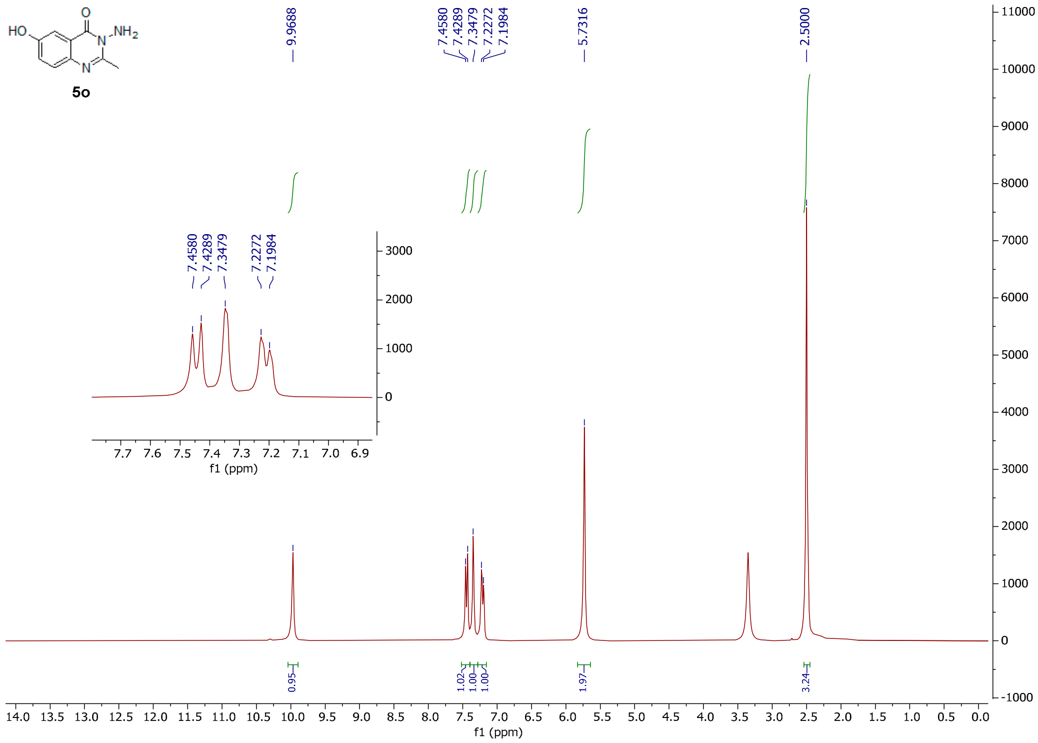

Figure S15: ^{1}H-NMR of 3-amino-6-hydroxy-2-methylquinazolin-4(3*H*)-one (**5o**)
